# Use of US Food and Drug Administration Expedited Drug Development and Review Programs by Orphan and Nonorphan Novel Drugs Approved From 2008 to 2021

**DOI:** 10.1001/jamanetworkopen.2022.39336

**Published:** 2022-11-01

**Authors:** Andrea N. Monge, Daniel W. Sigelman, Robert J. Temple, Harinder Singh Chahal

**Affiliations:** 1 Office of the Commissioner, US Food and Drug Administration, Silver Spring, Maryland; 2 Center for Drug Evaluation and Research, US Food and Drug Administration, Silver Spring, Maryland

## Abstract

**Importance:**

The US Food and Drug Administration (FDA) has 4 programs that can be used alone or in combination to expedite drug availability: Accelerated Approval, Breakthrough Therapy, Fast Track, and Priority Review. Drugs using these programs can include novel drugs, which do not contain a previously FDA-approved active moiety, and orphan drugs, intended for diseases or conditions affecting fewer than 200 000 people; to date, no comprehensive evaluation of how these programs have been used in combination has been published.

**Objective:**

To assess how often and in what combinations expedited programs are used in the development and review of approved novel biologics and small-molecule drugs, stratified by orphan drug status and indication.

**Design, Setting, and Participants:**

This cross-sectional study evaluated all novel drugs that were FDA approved between January 1, 2008, and December 31, 2021.

**Main Outcomes and Measures:**

The main outcome was the frequency with which expedited programs were used and in what combinations, stratified by orphan drug status and drug type (small molecule vs therapeutic biologic). The unit of analysis was the novel drug–indication pair because a drug can be approved for multiple indications, each of which may use a different expedited program or differ in orphan drug status.

**Results:**

The study included 581 novel drug–indication pairs approved during the 14-year study period; 252 (43.4%) were orphan drugs, 139 (23.9%) were therapeutic biologics, and 442 (76.1%) were small-molecule drugs. Use of at least 1 expedited program increased from 11 of 26 drug-indication pairs (42.3%) in 2008 to 41 of 55 (74.5%) in 2021. Of the 363 approved drug-indication pairs using at least 1 expedited program, 225 (62.0%) were orphan drugs; at least 1 expedited program was used by 97 of the 139 approved biologic drugs (69.8%) and by 266 of the 442 approved small-molecule drugs (60.2%). Eighty-two of the 581 novel drug–indication pairs (14.1%) used the Accelerated Approval Program; of those, 65 (79.3%) were oncology drugs and 70 (85.4%) had an orphan designation.

**Conclusions and Relevance:**

The study showed that use of the FDA’s expedited programs to bring novel drugs to market in the US increased from 2008 to 2021. The findings suggest that this trend is likely to continue.

## Key Points

**Question:**

How often and in what combinations are the 4 US Food and Drug Administration (FDA) programs for expedited development and review of orphan and nonorphan novel drugs used?

**Findings:**

In this cross-sectional study of 581 FDA-approved pairs of novel drugs and indications, use of expedited development and review programs increased from 42.3% of pairs in 2008 to 74.5% in 2021. Of approved drug-indication pairs using at least 1 expedited program, 62.0% were orphan drugs, 69.8% were biologics, and 60.2% were small-molecule drugs.

**Meaning:**

The findings suggest that expedited programs have an increasing role in bringing novel drugs to market in the US, especially orphan and biologic products.

## Teaser

This cross-sectional study examines how often and in what combinations 4 US Food and Drug Administration programs for expedited development and review of novel drugs were used among orphan and nonorphan biologics and small-molecule drugs approved between 2008 and 2021.

## Introduction

The US Food and Drug Administration (FDA) has 4 programs designed to expedite the availability of drugs and therapeutic biologics that substantially advance therapy (Table 1),^1^ all of which may be used alone or in combination. The first program, Fast Track designation, began in 1988 when, responding to the AIDS epidemic, the FDA established a new regulatory pathway recognizing that certain drugs may warrant expedited review procedures. Congress later codified the designation in the Food and Drug Administration Modernization Act of 1997.^2^ The Fast Track designation expedites drug development for serious conditions, addressing unmet medical needs, and it involves early communication with the FDA and review of applications in portions.^1^ The FDA can rescind this designation if a drug no longer meets designation requirements.^1^

**Table 1.** Overview of the FDA’s Expedited Drug Development and Review Programs

Program attribute	Fast Track	Accelerated Approval	Breakthrough Therapy	Priority Review
Year implemented	1988	1992	2012	1992
Promotes drug development	Yes	Yes	Yes	No
Rolling review^a^	Yes	No	Yes	No
Decreases application review time after FDA submission	No	No	No	Yes
Qualifying criteria for drugs	Treat a serious condition and address an unmet medical need or drug is a QIDP^b^	Treat a serious condition and provide a meaningful advantage over available therapies and may use a surrogate end point reasonably likely to predict clinical benefit or an intermediate clinical end point reasonably likely to predict an effect on irreversible morbidity or mortality	Treat a serious condition and preliminary clinical data demonstrate substantial improvement over available therapies	Treat a serious condition and if approved, would provide a significant improvement in safety or effectiveness over available therapies
Benefits to drug applicants	Expedited development and review; rolling review of application materials	Quicker market entry before completion of confirmatory trials	Intensive FDA guidance on efficient drug development, including meetings with senior reviewers; rolling review	Application reviewed in 6 mo compared with the 10-mo standard review
When to request program	With or after the IND but no later than the pre-BLA or pre-NDA submission meeting^c^	Applicants should discuss the possibility of accelerated approval with the FDA during development	With or after the IND but no later than the end-of-phase-2-trial meeting^d^	When application is submitted for FDA review
FDA response timelines	Within 60 calendar days of receiving the request	No specific timeline	Within 60 calendar days of receiving the request	Within 60 calendar days of receiving the application
Possible rescission of designation	Yes^e^	NA	Yes^e^	NA

Abbreviations: BLA, biologic licensing application; FDA, US Food and Drug Administration; IND, investigative new drug application; NA, not applicable; NDA, new drug application; QDIP, qualified infectious disease product.

^a^ Indicates that a drug company can submit completed sections of a BLA or an NDA for review by the FDA rather than waiting to complete the entire application before submission to the FDA for review.

^b^ A QDIP is an antibacterial or antifungal drug intended to treat serious or life-threatening infections in humans. This study did not distinguish QIDPs in the analysis, but some of the novel drugs included in this study received QIDP designation.

^c^ Pre-BLA and pre-NDA submission meetings are meetings with the FDA that may be requested by the drug applicant seeking to discuss filing and format issues before submitting an NDA or a BLA for FDA review.

^d^ The end-of-phase-2-trial meeting is a meeting between the FDA and drug developers with an IND after the completion of phase 2 trials to discuss options for trial designs, among other topics; to optimize dose selection for subsequent trials; and to improve the efficiency of drug development.

^e^ The FDA may rescind Fast Track or Breakthrough Therapy designations if the criteria for the respective programs are no longer met.

In October 1992, Congress passed the Prescription Drug User Fee Act, which established Priority Review designation for drugs that may provide a “significant improvement in the safety or effectiveness of the treatment, prevention, or diagnosis of a serious condition.”^1^ The FDA’s goal is to complete the review of drug applications meeting Priority Review requirements within 6 months instead of the standard 10 months.^1,2^ In December 1992, the FDA finalized a regulation for the Accelerated Approval pathway for drugs treating serious or life-threatening conditions with an unmet medical need; the FDA may approve these drugs based on surrogate end points that are “reasonably likely” to predict clinical benefit or based on intermediate clinical end points “other than survival or irreversible morbidity.”^1^ The Accelerated Approval pathway is contingent on an applicant’s agreement to conduct “adequate and well-controlled” postapproval studies to confirm clinical benefit.^3^ Congress codified this 20 years later in the Food and Drug Administration Safety and Innovation Act of 2012.^2^ This law also established the Breakthrough Therapy designation,^2^ which expedites development and review of drugs demonstrating substantial improvement over available therapy by providing for intensive early involvement of senior FDA managers.^1^ As with the Fast Track designation, the FDA can rescind the Breakthrough Therapy designation if the requirements are no longer met.^1^

Separately, under the Orphan Drug Act of 1983, the FDA can designate certain drugs and biologics intended for rare diseases or conditions (those affecting fewer than 200 000 people in the US) as orphan drugs.^4^ While not part of an expedited drug development or review program, the orphan drug designation primarily provides financial incentives for the development of drug indications likely to have a small market. Potential incentives include a 25% tax credit for applicable research and development expenditures, waived user fees for applications submitted to the FDA, and the potential for 7 years of exclusivity after approval.^4^ Orphan drugs may also use expedited programs during their development or FDA review, should they qualify.^5^

Previous literature has examined trends in overall use of expedited programs.^6,7,8,9^ However, to our knowledge, no publication has comprehensively evaluated how the FDA and applicants use these programs in combination (ie, “stacked”). Similarly, while other research has examined the trends in orphan drug approvals for both biologics and small-molecule drugs,^5,10,11,12^ published data on how orphan vs nonorphan drugs use the 4 expedited programs are sparse. In this study, we examined use of the programs alone or in combination to approve novel biologics and small-molecule drugs by specific drug indication and orphan drug designation status from 2008 to 2021.

## Methods

### Study Design and Data Source

We conducted a cross-sectional study of all novel small-molecule drugs (new drug applications) and therapeutic biologics (biologic license applications) approved by the FDA’s Center for Drug Evaluation and Research between January 1, 2008, and December 31, 2021. The study included the initial new drug and biologic license applications (ie, the original applications). Any supplemental applications to add new drug indications were excluded from this analysis. Although the Breakthrough Therapy designation was initiated in 2012, this study included data starting from 2008, the earliest full year of data available to us, to provide the most comprehensive analysis on the use of expedited drug development and review programs. This cross-sectional study did not involve clinical data or human participants; therefore, it was not submitted for ethics review. The study adhered to the Strengthening the Reporting of Observational Studies in Epidemiology (STROBE) reporting guideline.

Data on approved novel drugs were collected from an internal FDA database, the Data Analysis and Search Host. This database provided, at the drug indication level, drug-specific attributes such as the drug name, type of drug (small molecule vs therapeutic biologic), approval date, expedited program(s) used, first-in-class status, and first-approved-in-the-US status. We developed methods to assign therapeutic areas to the novel drugs.

### Study Definition of a Novel Drug

A novel drug is one that does not contain a previously FDA-approved active moiety.^13^ Typically, the FDA reports provide novel drug information at the active ingredient level; however, this study used each FDA-approved indication in the drug label as a separate unit of analysis to provide the most granular level of information. Because a drug can be initially approved for more than 1 indication and individual indications may have used different expedited programs or may differ in orphan drug status, our study of the use of expedited drug development and review programs considered each approved drug-indication pair as a separate novel drug.

For example, umbralisib was initially approved for 2 indications: (1) treatment of adult patients with relapsed or refractory marginal zone lymphoma (MZL) and (2) treatment of adult patients with relapsed or refractory follicular lymphoma (FL). Both indications were for rare diseases that qualified the drug for an orphan designation; however, while the MZL indication received Priority Review and Breakthrough Therapy designation, the FL received neither. To account for this variability, we analyzed each indication as a distinct novel drug–indication pair (umbralisib-MZL and umbralisib-FL). In another example, brexpiprazole, initially approved for 2 indications (major depressive disorder and schizophrenia), was also analyzed as 2 drugs for this study, although the indications did not vary by orphan status or use of expedited drug development or review programs. We consistently applied this method to all drugs in the sample.

### Use of Expedited Programs

To assess the use and stacking of expedited programs, we first determined whether a drug-indication pair used 1 or more of the 4 expedited programs during its development or review. We then ascertained whether either the Fast Track or the Breakthrough Therapy designations were rescinded after being initially granted. For those rescinded, we removed the designations from the affected drug-indication pair. We analyzed the remaining pairs’ use and stacking of expedited programs. We conducted a focused analysis of drug-indication pairs receiving Accelerated Approval because it is the only pathway by which drugs may receive FDA authorization to market based on surrogates or intermediate clinical end points and is currently the subject of debate among the medical community.

### Categorization of Therapeutic Areas

After a detailed review of a novel drug’s mechanism(s) of action and indication(s) and based on their clinical experience, 2 investigators (A.N.M. and H.S.C.) determined the therapeutic area to which each drug should be assigned; any disagreements were resolved by consensus. Because uses of some drugs may involve overlapping therapeutic areas, other clinicians may assign the same drug to another therapeutic area. For research purposes, we sought to categorize drugs by therapeutic area based on their clinical effects. For example, while drugs for treatment of Duchenne muscular dystrophy could be classified under genetic disorders, we classified them under neurology. Similarly, although capsaicin is indicated for the management of neuropathic pain and can be considered an analgesic, we classified it under neurology because the indication is for pain associated with postherpetic neuralgia. Our study classified defibrotide sodium, indicated for the treatment of sinusoidal obstruction syndrome after hematopoietic stem-cell transplant, as a transplant drug, but others might consider it an oncologic drug. Because we used broad therapeutic categories, any minor differences in therapeutic area categorization were unlikely to affect our findings.

### Statistical Analysis

The unit of analysis was the novel drug–indication pair. We conducted descriptive statistics on novel drug attributes, including first-in-class status and first-approved-in-the-US status. We also analyzed how many drug-indication pairs used at least 1 program, 2 or more programs, 3 or more programs, or all 4 programs simultaneously. We then analyzed the combination in which the 4 programs were used to determine how they were stacked by each drug-indication pair.

The results were stratified by drug status (orphan vs nonorphan) and type of drug (small molecule vs therapeutic biologic). By year of drug approval, we also analyzed the trends in use of the expedited programs, stratified by orphan drug status. Finally, we analyzed, by therapeutic area, the frequency with which expedited programs were used and in what combinations. Data analyses were conducted using R, version 4.1 (R Project for Statistical Computing).

## Results

### Overview of Findings

The study sample included 581 novel drug–indication pairs approved during the 14-year study period, of which 252 (43.4%) were designated as orphan drugs and 329 (56.6%) were not (Table 2). Of the 581 drug-indication pairs, 139 (23.9%) were therapeutic biologics and 442 (76.1%) were small-molecule drugs. Among the 139 biologic and 442 small-molecule drug–indication pairs, 75 (54.0%) and 177 (40.0%), respectively, were designated as orphan drugs.

**Table 2.** Use of Expedited Drug Development Programs by Novel Drug–Indication Pairs Approved From 2008 to 2021, Stratified by Drug Type and Orphan Drug Status

	Drug-indication pairs, No./total No. (%)								
	All novel drugs			Therapeutic biologics^a^			Small-molecule drugs^b^		
	All^c^	Orphan^d^	Nonorphan^d^	All^c^	Orphan^d^	Nonorphan^d^	All^c^	Orphan^d^	Nonorphan^d^
Total	581/581 (100)	252/581 (43.4)	329/581 (56.6)	139/581 (23.9)	75/139 (54.0)	64/139 (46.0)	442/581 (76.1)	177/442 (40.0)	265/442 (60.0)
First in class^e^	215/581 (37.0)	128/215 (59.5)	87/215 (40.5)	72/139 (51.8)	47/72 (65.3)	25/72 (34.7)	143/442 (32.4)	81/143 (56.6)	62/143 (43.4)
First approved in the US^f^	400/581 (68.8)	182/400 (45.5)	218/400 (54.5)	100/139 (71.9)	59/100 (59.0)	41/100 (41.0)	300/442 (67.9)	123/300 (41.0)	177 /300 (59.0)
Expedited drug development programs used, No.									
0	218/581 (37.5)	27/218 (12.4)	191/218 (87.6)	42/139 (30.2)	7/42 (16.7)	35/42 (83.3)	176/442 (39.8)	20/176 (11.4)	156/176 (88.6)
≥1	363/581 (62.5)	225/363 (62.0)	138/363 (38.0)	97/139 (69.8)	68/97 (70.1)	29/97 (29.9)	266/442 (60.2)	157/266 (59.0)	109/266 (41.0)
≥2	257/581 (44.2)	178/257 (69.3)	79/257 (30.7)	73/139 (52.5)	56/73 (76.7)	17/73 (23.3)	184/442 (41.6)	122/184 (66.3)	62/184 (33.7)
≥3	97/581 (16.7)	76/97 (78.4)	21/97 (21.6)	31/139 (22.3)	22/31 (71.0)	9/31 (29.0)	66/442 (14.9)	54/66 (81.8)	12/66 (18.2)
4	13/581 (2.2)	11/13 (84.6)	2/13 (15.4)	7/139 (5.0)	5/7 (71.4)	2/7 (28.6)	6/442 (1.4)	6/6 (100)	0
Use of specific expedited programs alone or in combination^g^									
Accelerated Approval	82/581 (14.1)	70/82 (85.4)	12/82 (14.6)	24/139 (17.3)	15/24 (62.5)	9/24 (37.5)	58/442 (13.1)	55/58 (94.8)	3/58 (5.2)
Breakthrough Therapy^h^	115/581 (19.8)	88/115 (76.5)	27/115 (23.5)	44/139 (31.7)	34/44 (77.3)	10/44 (22.7)	71/442 (16.1)	54/71 (76.1)	17/71 (23.9)
Fast Track	203/581 (34.9)	126/203 (62.1)	77/203 (37.9)	53/139 (38.1)	42/53 (79.2)	11/53 (20.8)	150/442 (33.9)	84/150 (56.0)	66/150 (44.0)
Priority Review	330/581 (56.8)	206/330 (62.4)	124/330 (37.6)	87/139 (62.6)	60/87 (69.0)	27/87 (31.0)	243/442 (55.0)	146/243 (60.1)	97 /243 (39.9)
Stacked use of expedited programs									
Only Accelerated Approval	2/581 (0.3)	2/2 (100)	0	0	NA	NA	2/442 (0.5)	2/2 (100)	0
Only Breakthrough Therapy	1/581 (0.2)	1/1 (100)	0	1/139 (0.7)	1/1 (100)	0	0	NA	NA
Only Fast Track	25/581 (4.3)	11/25 (44.0)	14/25 (56.0)	8/139 (5.8)	6/8 (75.0)	2/8 (25.0)	17/442 (3.8)	5/17 (29.4)	12/17 (70.6)
Only Priority Review	78/581 (13.4)	33/78 (42.3)	45/78 (57.7)	15/139 (10.8)	5/15 (33.3)	10/15 (66.7)	63/442 (14.3)	28/63 (44.4)	35/63 (55.6)
Accelerated Approval and Fast Track	4/581 (0.7)	4/4 (100)	0	0	NA	NA	4/442 (0.9)	4/4 (100)	0
Accelerated Approval and Priority Review	13/581 (2.2)	12/13 (92.3)	1/13 (7.7)	3/139 (2.2)	2/3 (66.7)	1/3 (33.3)	10/442 (2.3)	10/10 (100)	0
Breakthrough Therapy and Fast Track	1/581 (0.2)	1/1 (100)	0	1/139 (0.7)	1/1 (100)	0	0	NA	NA
Breakthrough Therapy and Priority Review	36/581 (6.2)	28/36 (77.8)	8/36 (22.2)	14/139 (10.1)	12/14 (85.7)	2/14 (14.3)	22/442 (5.0)	16/22 (72.7)	6/22 (27.3)
Fast Track and Priority Review	106/581 (18.2)	57/106 (53.8)	49/106 (46.2)	24/139 (17.3)	19/24 (79.2)	5/24 (20.8)	82/442 (18.6)	38/82 (46.3)	44/82 (53.7)
Accelerated Approval, Breakthrough Therapy, and Priority Review	30/581 (5.2)	23/30 (76.7)	7/30 (23.3)	11/139 (7.9)	6/11 (54.5)	5/11 (45.5)	19/442 (4.3)	17/19 (89.5)	2/19 (10.5)
Accelerated Approval, Fast Track, and Priority Review	20/581 (3.4)	18/20 (90.0)	2/20 (10.0)	3/139 (2.2)	2/3 (66.7)	1/3 (33.3)	17/442 (3.8)	16/17 (94.1)	1/17 (5.9)
Breakthrough Therapy, Fast Track, and Priority Review	34/581 (5.9)	24/34 (70.6)	10/34 (29.4)	10/139 (7.2)	9/10 (90.0)	1/10 (10.0)	24/442 (5.4)	15/24 (62.5)	9/24 (37.5)
Accelerated Approval, Breakthrough Therapy, Fast Track, and Priority Review	13/581 (2.2)	11/13 (84.6)	2/13 (15.4)	7/139 (5.0)	5/7 (71.4)	2/7 (28.6)	6/442 (1.4)	6/6 (100)	0
Therapeutic area^i^									
Oncology	150/581 (25.8)	110/150 (73.3)	40/150 (26.7)	44/139 (31.7)	30/44 (68.2)	14/44 (31.8)	106/442 (24.0)	80/106 (75.5)	26/106 (24.5)
Infectious diseases	71/581 (12.2)	22/71 (31.0)	49/71 (69.0)	6/139 (4.3)	5/6 (83.3)	1/6 (16.7)	65/442 (14.7)	17/65 (26.2)	48/65 (73.8)
Neurology	63/581 (10.8)	29/63 (46.0)	34/63 (54.0)	16/139 (11.5)	5/16 (31.2)	11/16 (68.8)	47/442 (10.6)	24/47 (51.1)	23/47 (48.9)
Endocrinology, diabetes, and metabolism	59/581 (10.2)	32/59 (54.2)	27/59 (45.8)	26/139 (18.7)	16/26 (61.5)	10/26 (38.5)	33/442 (7.5)	16/33 (48.5)	17/33 (51.5)
Gastroenterology	30/581 (5.2)	7/30 (23.3)	23/30 (76.7)	3/139 (2.2)	0	3/3 (100)	27/442 (6.1)	7/27 (25.9)	20/27 (74.1)
Dermatology	27/581 (4.6)	1/27 (3.7)	26/27 (96.3)	11/139 (7.9)	0	11/11 (100)	16/442 (3.6)	1/16 (6.2)	15/16 (93.8)
Diagnostics: medical imaging	26/581 (4.5)	5/26 (19.2)	21/26 (80.8)	0	NA	NA	26/442 (5.9)	5/26 (19.2)	21/26 (80.8)

Abbreviation: NA, not applicable.

^a^ Biologic license applications.

^b^ New drug applications.

^c^ Percentages are shown for the column.

^d^ Percentages are shown for the row.

^e^ The drug was the first with a novel active moiety in the drug class to be approved by the US Food and Drug Administration.

^f^ The drug moiety was approved in the US before another country.

^g^ Each novel drug–indication pair may use multiple expedited programs. In this analysis, the sum of the expedited programs used was greater than the total number of novel drug–indication pairs in the analysis; thus, the sum of the column percentages exceeds 100%.

^h^ Breakthrough Therapy programs started in 2012, 4 years after the start of our study period.

^i^ For readability, the table only shows the 8 therapeutic areas that had 25 or more novel drug–indication pairs, accounting for 448 of the drugs in this analysis (77.1%). The 12 other therapeutic areas, accounting for 133 of the novel drug–indication pairs in this analysis (22.9%), are not shown.

### Use and Stacking of Expedited Programs

Overall, of 581 drug-indication pairs, 363 (62.5%) used at least 1 expedited program, 257 (44.2%) used 2 or more programs, 97 (16.7%) used 3 or more programs, and 13 (2.2%) used all 4 programs; 218 (37.5%) did not use any expedited programs (Table 2). Ninety-seven of the 139 biologic drug–indication pairs (69.8%) and 266 of the 442 small-molecule drug–indication pairs (60.2%) used at least 1 program.

Priority Review was the most frequently used program; 330 drug-indication pairs (56.8%) used it either alone or in combination with other programs. This was followed by Fast Track (203 pairs [34.9%]), Breakthrough Therapy (115 pairs [19.8%]), and Accelerated Approval (82 pairs [14.1%]) (Table 2).

**Figure 1.**
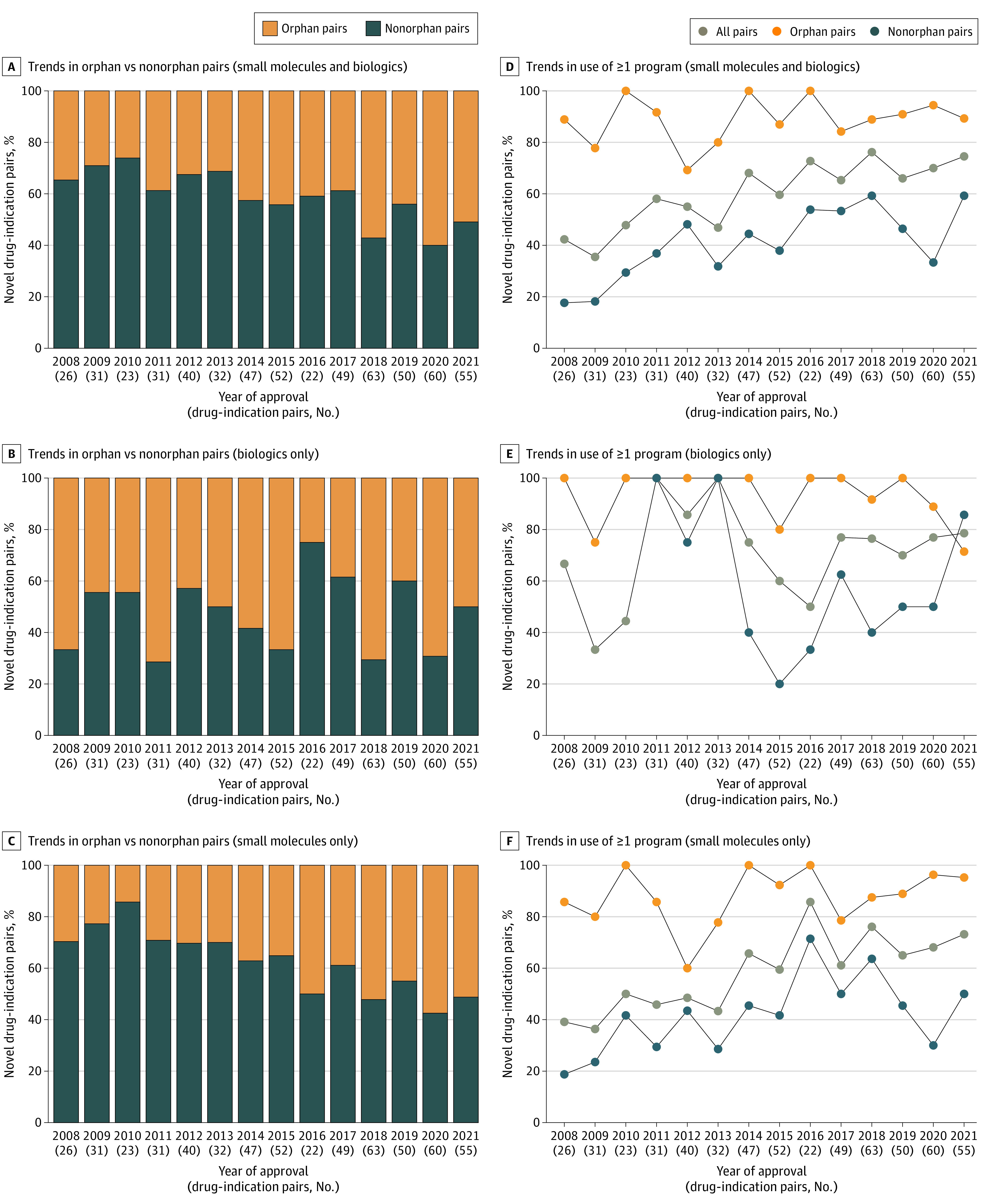
Percentage of Approved Orphan and Nonorphan Novel Drug–Indication Pairs Using at Least 1 Expedited Drug Development and Review Program From 2008 to 2021

Use of at least 1 expedited program among novel drug–indication pairs increased from 11 of 26 pairs (42.3%) in 2008 to 41 of 55 (74.5%) in 2021. In every year from 2008 to 2021 except 2009 and 2010, more than 50% of biologics used at least 1 expedited review program (Figure 1). Use of at least 1 expedited review program among small-molecule drugs increased during the study period. Breaking this down by orphan and nonorphan small-molecule drug–indication pairs, the increase appears to have been prompted by nonorphan drugs. In contrast, use of at least 1 expedited review program by orphan small-molecule drug–indication pairs remained comparatively constant at more than 75% every year except 2012 (Figure 1).

The expedited programs were stacked in various combinations (Table 2). The most common program combination was Fast Track with Priority Review, which was used by 106 of the 581 drug-indication pairs (18.2%) (Table 2). The combination of Breakthrough Therapy with Fast Track was the least used (1 drug-indication pair [0.2%]).

### Orphan Drugs

Orphan drug–indication pairs had greater use of each expedited program compared with nonorphan pairs. Orphan drug–indication pairs also most frequently used stacked programs: of the 363 drug-indication pairs that used at least 1 expedited program, 225 (62.0%) had an orphan drug designation; of the 257 pairs using at least 2 programs, 178 (69.3%) had an orphan drug designation; of the 97 pairs using at least 3 programs, 76 (78.4%) had an orphan drug designation; and of the 13 pairs using all 4 programs, 11 (84.6%) had an orphan drug designation (Table 2). Of the 97 therapeutic biologic–indication pairs using at least 1 expedited program, 68 (70.1%) had an orphan drug designation, whereas only 157 of the 266 small-molecule drug–indication pairs (59.0%) using at least 1 expedited program had an orphan drug designation.

Expedited program use was higher among orphan drug–indication pairs, with a minimal relative increase over time from 8 of 9 pairs (88.9%) using at least 1 expedited program in 2008 to 25 of 28 pairs (89.3%) in 2021 (Figure 1). By contrast, the relative increase in expedited program use was larger among nonorphan drug–indication pairs from 3 of 17 pairs (17.6%) using at least 1 expedited program in 2008 to 16 of 27 pairs (59.3%) in 2021 (Figure 1).

### Use of Expedited Programs Among Therapeutic Areas

**Figure 2.**
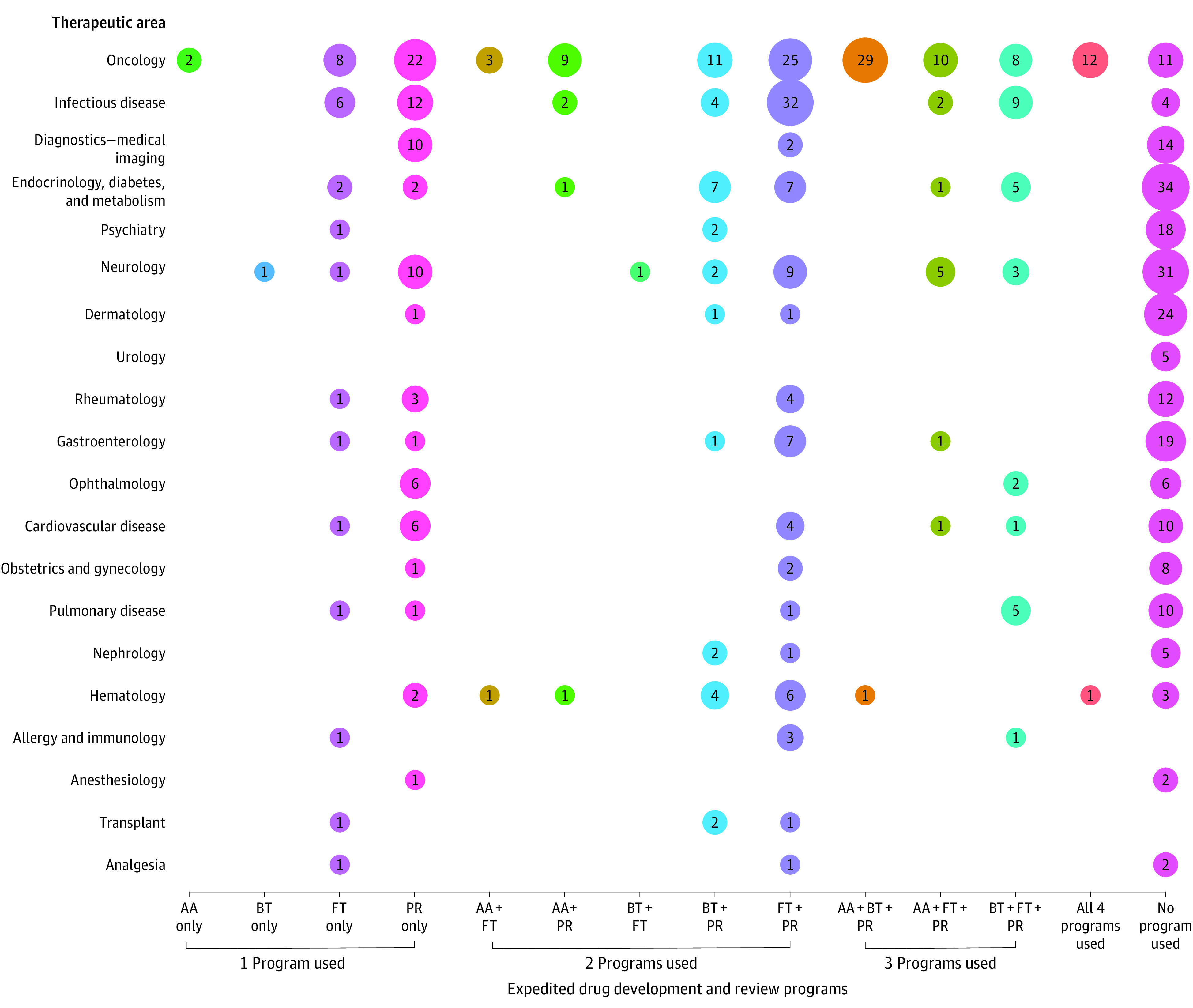
Use of Expedited Drug Development and Review Programs Among Novel Drug–Indication Pairs Approved From 2008 to 2021, by Therapeutic Area Numbers in the circles are the number of novel drug–indication pairs that used each program type; larger circles signify greater numbers of pairs. AA indicates Accelerated Approval pathway; BT, Breakthrough Therapy designation; FT, Fast Track designation; and PR, Priority Review designation.

The use of expedited programs, including in combinations, highly varied by therapeutic area. Some therapeutic areas heavily relied on expedited programs, while others used none (Figure 2). For example, 139 of 150 oncology drug-indication pairs (92.7%) and 67 of 71 infectious disease pairs (94.4%) used at least 1 program (Figure 2). In contrast, none of the 5 approved urology drug-indication pairs used expedited programs.

### Use of Accelerated Approval

In total, 82 of the 581 novel drug–indication pairs (14.1%) used the Accelerated Approval program. Of the 82 drug-indication pairs using Accelerated Approval, 65 (79.3%) were oncology drugs (Table 3). The therapeutic area with the next-highest use of Accelerated Approval was neurology, with 5 of the 82 drug-indication pairs (6.1%). Overall, 65 of 150 oncology drug-indication pairs (43.3%) used Accelerated Approval vs 17 of 481 nononcology pairs (3.5%). Furthermore, among the drug-indication pairs using Accelerated Approval, 70 of 82 (85.4%) had an orphan designation; of those, 55 (78.6%) were oncology drugs.

**Table 3.** Therapeutic Areas Using the Accelerated Approval Pathway Among Novel Drug–Indication Pairs Approved From 2008 to 2021

Therapeutic area	Drug-indication pairs using Accelerated Approval, No./total No. (%)								
	All novel drugs			Therapeutic biologics^a^			Small-molecule drugs^b^		
	All^c^	Orphan^d^	Nonorphan^d^	All^c^	Orphan^d^	Nonorphan^d^	All^c^	Orphan^d^	Nonorphan^d^
Total	82/82 (100)	70/82 (85.4)	12/82 (14.6)	24/82 (29.3)	15/24 (62.5)	9/24 (37.5)	58/82 (70.7)	55/58 (94.8)	3/58 (5.2)
Oncology	65/82 (79.3)	55/65 (84.6)	10/65 (15.4)	22/24 (91.7)	14/22 (63.6)	8/22 (36.4)	43/58 (74.1)	41/43 (95.3)	2/43 (4.7)
Neurology	5/82 (6.1)	4/5 (80.0)	1/5 (20.0)	1/24 (4.2)	0	1/1 (100)	4/58 (6.9)	4/4 (100)	0
Hematology	4/82 (4.9)	4/4 (100)	0	1/24 (4.2)	1/1 (100)	0	3/58 (5.2)	3/3 (100)	0
Infectious diseases	4/82 (4.9)	3/4 (75.0)	1/4 (25.0)	0	NA	NA	4/58 (6.9)	3/4 (75.0)	1/4 (25.0)
Endocrinology, diabetes, and metabolism	2/82 (2.4)	2/2 (100)	0	0	NA	NA	2/58 (3.4)	2/2 (100)	0
Cardiovascular disease	1/82 (1.2)	1/1 (100)	0	0	NA	NA	1/58 (1.7)	1/1 (100)	0
Gastroenterology	1/82 (1.2)	1/1 (100)	0	0	NA	NA	1/58 (1.7)	1/1 (100)	0

Abbreviation: NA, not applicable.

^a^ Biologic license applications.

^b^ New drug applications.

^c^ Percentages are shown for the column.

^d^ Percentages are shown for the row.

## Discussion

In this study, use of expedited programs alone and in combination for novel drug development and approval was common among orphan and nonorphan drug products and increased over time, highlighting the integral roles of these programs in bringing novel drugs to market. We found that orphan drug products were approved proportionally more often with 1 or more expedited programs than were nonorphan drugs. This is consistent with orphan drugs being more likely to target rare conditions with an unmet medical need. Our findings highlight the central role of expedited programs in affording patients with unmet medical needs timely access to orphan drugs.

Although direct comparison with existing literature was not possible because this study evaluated expedited program use at the indication level during a 14-year period, in general, our findings are consistent with observations from previous studies. As with earlier studies, we observed increasing use over time of expedited programs,^6^ with Priority Review being the most frequently used and Accelerated Approval the least frequently used program.^6,7^ Furthermore, we noted, as observed in prior publications, that drugs with oncology indications were approved through 1 or more expedited programs more often than were drugs in other therapeutic areas.^7,14^

The use of expedited programs is likely to continue to increase over time. During the past 3 decades, the number of drugs receiving FDA orphan drug designation increased 4 fold,^12^ increasing the pool of drugs disproportionately likely to qualify for expedited programs. This increase in orphan drug designations is primarily associated with oncology indications (which can be divided further into more specific genetic subtypes that can occur in 200 000 patients or fewer),^6^ accompanied by a noticeable increase in pediatric conditions such as cystic fibrosis, Duchenne muscular dystrophy, and sickle cell disease.^12^ Furthermore, in our study, 85.4% of all drug-indication pairs using Accelerated Approval had an orphan designation. With an increase in more conditions for narrowly defined patient populations, it appears likely that use of expedited programs such as Accelerated Approval by orphan drugs will increase.

During the study period, approved biologics, although fewer in absolute number, generally used the expedited programs more than small-molecule drugs did. This finding reflects, in part, advances in medical innovation that are bringing an array of new types of therapeutics to the forefront. Compared with small-molecule drugs, biologics are generally technologically innovative, structurally complex products derived from either human, animal, or microorganism living material.^15^ Although the objective of the expedited review programs is not to facilitate medical innovation, the programs may indirectly provide an avenue for advanced treatment technologies to reach patients. In this study, 69.8% of approved novel biologic–indication pairs used at least 1 expedited program.

In addition, there has been increasing scrutiny of the level of evidence that the FDA requires under these expedited programs. While evaluation of the evidence supporting approved novel drugs was outside the scope of our analyses, overall, the literature suggests that drugs approved using expedited programs are based on fewer and smaller clinical studies than are those not using any expedited review program^16^ and are associated with a higher likelihood of FDA safety actions after market entry.^16,17^ Expressions of efficacy and/or safety concerns regarding Accelerated Approval drugs, for which clinical benefit requires confirmation in postapproval studies, have been especially prominent in the literature.^18,19,20^ Stakeholders have recommended enhanced FDA enforcement of postmarket study requirements for drugs approved under the Accelerated Approval pathway.^21^ Congress is considering changes to the Accelerated Approval program,^22,23,24^ such as strengthening the requirement to conduct appropriate confirmatory studies and authorizing the FDA to require that postmarket studies be underway before issuing marketing authorization.^23^

Our study showed that novel oncology drug indications were more likely to have been approved under the Accelerated Approval pathway than were indications assigned to other therapeutic areas. In recent years, the FDA has focused particular attention on the outcomes of confirmatory studies conducted to confirm the clinical benefit of several such indications as part of its ongoing “industry-wide evaluation of accelerated approvals in oncology in which confirmatory trials did not confirm clinical benefit.”^25,26,27^ Since 2011, 21 cancer-specific indications initially granted accelerated approval have been withdrawn; of these, 61.9% were withdrawn between February 2021 and May 2022.^28^ Such ongoing assessments may help balance timely therapy availability for serious conditions against the need to minimize exposure to drugs with unverified clinical benefit.^26^ The complex interaction between the programs and their implications for the standards of evidence required for approval are beyond the scope of this study but need to be further understood to ensure that any changes to these programs continue to afford patient access to life-saving drugs with appropriate safeguards.

### Limitations

This study has limitations. First, it did not evaluate any causal effects of the use of expedited programs or orphan drug status on drug approval or the drugs’ safety and efficacy. Second, this study did not assess whether the expedited programs reduced drug development or approval time. While previous literature has evaluated this question,^29,30,31^ it may be valuable to examine a larger time frame of approved drugs with and without expedited programs, including by specific indications and orphan drug status, to further elucidate the effect, if any, these programs have on drug development. Third, an analysis of whether more advanced treatment options are using expedited programs was not possible in this study design but warrants further examination. Fourth, the study findings cannot be extrapolated to other types of FDA medical products that may use other schemes for expedited development or review.

## Conclusions

This study found that use of the FDA’s expedited programs to bring orphan and nonorphan therapeutic biologics and small-molecule novel drugs to the US market increased from 2008 to 2021. The findings suggest that this trend is likely to continue.
