# Errors in Figure 1

**DOI:** 10.1001/jamanetworkopen.2023.2158

**Published:** 2023-02-20

In the Original Investigation titled “Use of US Food and Drug Administration Expedited Drug Development and Review Programs by Orphan and Nonorphan Novel Drugs Approved From 2008 to 2021,”^1^ published November 1, 2022, the numbers in parentheses on the x-axis were incorrect in Figure 1B, C, E, and F. This article has been corrected.^1^
